# Supplementary material for: High-throughput sequencing of SARS-CoV-2 in wastewater provides insights into circulating variants
Source: medRxiv. 2021 Jan 25:2021.01.22.21250320. Preprint. [Version 1] doi: 10.1101/2021.01.22.21250320 (PMC7836124; doi:10.1101/2021.01.22.21250320)
Supplement: Supplement 2 — Supplementary Figure 1: Wastewater sampling catchments in Louisville (Kentucky), Sites 1 and 7 represent collection sites of hospitals and Site 9 is a sewer district facility. [file media-2.pdf]

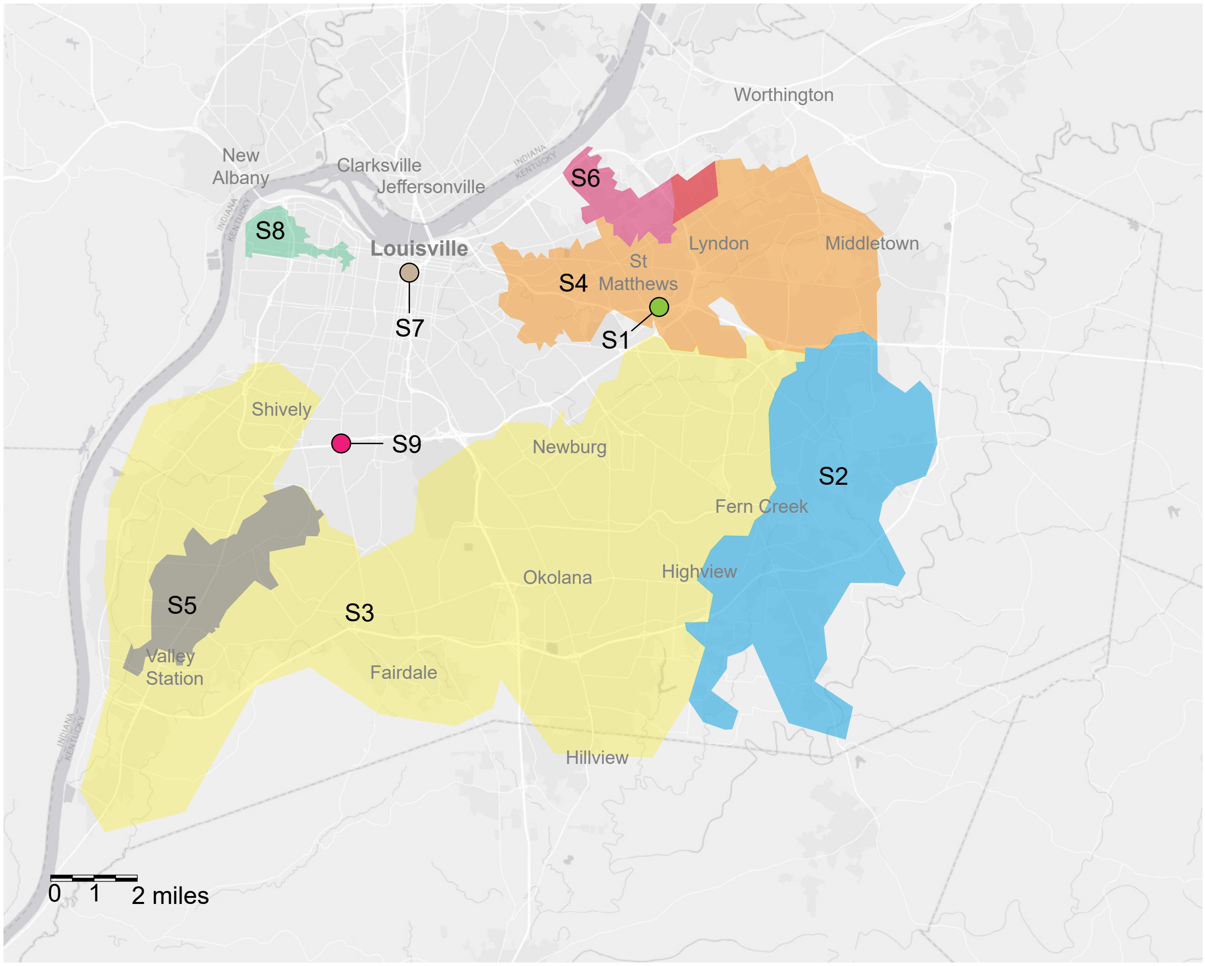

Supplementary Figure 1: Wastewater sampling catchments in Louisville (Kentucky), Sites 1 and 7 represent collection sites of hospitals and Site 9 is a sewer district facility.
